# Preparation and Characterization of Microsphere ZnO ALD Coating Dedicated for the Fiber-Optic Refractive Index Sensor

**DOI:** 10.3390/nano9020306

**Published:** 2019-02-23

**Authors:** Paulina Listewnik, Marzena Hirsch, Przemysław Struk, Matthieu Weber, Mikhael Bechelany, Małgorzata Jędrzejewska-Szczerska

**Affiliations:** 1 Department of Metrology and Optoelectronics, Faculty of Electronics, Telecommunications and Informatics, Gdańsk University of Technology, 11/12 Narutowicza Street, 80-233 Gdańsk, Poland; paulist@o2.pl (P.L.); marpluta1@student.pg.gda.pl (M.H.); 2 Department of Optoelectronics, Faculty of Electrical Engineering, Silesian University of Technology, 2 Krzywoustego Street, 44-100 Gliwice, Poland; 3 Institut Européen des Membranes (ENSCM, UMR CNRS 5635), Univ. Montpellier, Place Eugène Bataillon, 34095 Montpellier, France; matthieu.weber@umontpellier.fr (M.W.); mikhael.bechelany@umontpellier.fr (M.B.)

**Keywords:** ZnO, atomic layer deposition, coating, microsphere, fiber-optic sensors, refractive index

## Abstract

We report the fabrication of a novel fiber-optic sensor device, based on the use of a microsphere conformally coated with a thin layer of zinc oxide (ZnO) by atomic layer deposition (ALD), and its use as a refractive index sensor. The microsphere was prepared on the tip of a single-mode optical fiber, on which a conformal ZnO thin film of 200 nm was deposited using an ALD process based on diethyl zinc (DEZ) and water at 100 °C. The modified fiber-optic microsphere was examined using scanning electron microscopy and Raman spectroscopy. Theoretical modeling has been carried out to assess the structure performance, and the performed experimental measurements carried out confirmed the enhanced sensing abilities when the microsphere was coated with a ZnO layer. The fabricated refractive index sensor was operating in a reflective mode of a Fabry–Pérot configuration, using a low coherent measurement system. The application of the ALD ZnO coating enabled for a better measurement of the refractive index of samples in the range of the refractive index allowed by the optical fiber. The proof-of-concept results presented in this work open prospects for the sensing community and will promote the use of fiber-optic sensing technologies.

## 1. Introduction

Fiber-optic sensing devices present enormous potential, as they benefit from low-cost manufacturing, while maintaining high sensitivity and robustness. Nowadays, advanced fabrication techniques allow the incorporation of various nanomaterials for the tuning of sensing devices, including fiber-optic sensors [1,2,3,4]. Fiber-optic sensing devices can find application in many different fields of science such as chemistry (composition and content of various solutions) [1,2,5,6], biology [7,8,9,10] and physics [11,12,13,14]. Fiber-optics based sensors present a number of advantages, including high sensitivity, the ability to be used in demanding environments (narrow spaces, hazardous areas), as well as immunity to electromagnetic noise during operation [15,16]. Furthermore, the properties of fiber-optic sensors can easily be tuned by modifying their design with optical coatings. 

ZnO is an oxide semiconductor material presenting a wide energy band gap of ~3.3 eV [17]. The physical properties of ZnO, including the optical properties, strongly vary with the deposition technology used for its preparation. For sensing applications, ZnO films deposited as continuous layers with low surface roughness and/or in the form of nanoporous structures are morphologies which are particularly desired [18,19]. Concerning the optical properties, the refractive index of ZnO is around 2, and this semiconductor is optically transparent for the light above the absorption edge of 380 nm [20,21,22]. Due to these physical properties, ZnO coatings can be applied for a wide range of applications in sensing devices, for example for the detection of selected molecules in gases and liquids, and for the measurement of refractive indices [19,23,24,25,26,27,28]. 

One upcoming deposition technology that can be applied for the preparation of high quality ZnO nanomaterials is atomic layer deposition (ALD). This vapor phase deposition route is typically used for the synthesis of thin films with a thickness controllable at the (sub)nanometer scale. In fact, ALD enables the preparation of thin films and nanoparticles with controlled dimensions at the nanoscale on high aspect ratio substrates and is thus particularly appropriate for the coating of challenging substrates such as optical fibers [29,30,31,32,33].

Fiber-optic structures, such as microspheres are applied in the sensing systems. Most of them are coupled with fiber tapers and utilize the concept of Whispering Gallery Mode (WGM) in order to incite resonance. Successful applications of those sensors have been shown in biosensing and measurement of physical quantities (like temperature) [10,34]. To the best knowledge of the authors, so far none of the research uses fiber-optic microspheres as a low-coherence standalone refractive index sensor.

In this work, we report for the first time the modification of a fiber-optic microsphere-based sensor with a thin ZnO layer prepared by ALD. We also investigate the application of this ALD modified optical fiber as a refractive index sensor. Theoretical modeling is carried out to assess the structure performance, and experimental measurements are achieved to assess the sensing abilities of the new device. 

**Figure 1:**
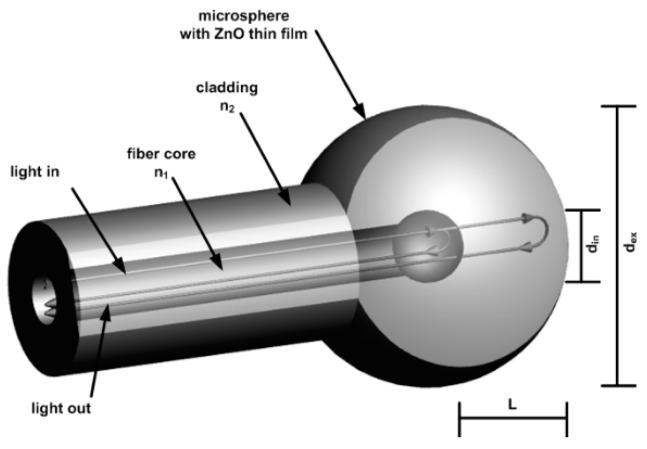
Schematic model of the microsphere with 200 nm ZnO thin film deposited on the surface, where: *d_in_*—internal diameter of the sphere, *d_ex_*—external diameter of the sphere, *L*—cavity length, *n_i_*—refractive index.

The refractive index sensing principle is based on the measurement of the light reflected from the external surface of the fiber-optic microsphere (Figure 1). In the case of the microsphere, there is also a weak reflection originating from the boundary between the core and the cladding material. The reflected beams interfere, creating a low finesse intrinsic Fabry–Pérot interferometer with a fixed cavity. The reflection of the core–-cladding interface is constant, but the value of the reflection coefficient of the sphere surface is highly dependent on the refractive index of the external medium. As its value is close to the refractive index value of the fiber cladding, the reflection decreases toward zero. The coating of the sphere surface by a thin film of transparent, high refractive index material like ZnO allows modification of the reflection function and shifts that transition point to the higher value of the surrounding refractive index [35,36]. Graphical visualization of the sensor structure is presented in Figure 1.

## 2. Materials and Methods

The fiber-optic sensing device fabricated in this work is based on a single-mode optical fiber SMF-28, purchased from Thorlabs (Newton, NJ, USA). The microsphere is formed on the tip using the optical fiber fusion splicer (FSU 975, Ericsson Network Technologies AB, Stockholm, Sweden). The splicer produces an electronic arc of, in this case, 14.9 mA and a three-step pull, each of a different duration. To obtain the sphere with a diameter of about 240 µm optical fiber was pulled for 3 s, collectively. 

All ALD depositions have been carried out in a custom-built ALD reactor described elsewhere [37,38]. Diethyl zinc (DEZ) precursor was purchased from Sigma Aldrich and used as received. The co-reactant was millipore water. The substrates used were p-type (100) silicon wafers (MEMC Korea Company, Cheonan, Korea) and SMF-28 optical fibers (Thorlabs, Newton, NJ, USA). To remove the organic contaminants, the substrates were pre-cleaned in acetone, ethanol and de-ionized water for 5 min in an ultrasonic bath before the depositions. ALD of ZnO was performed using sequential exposures of DEZ and H_2_O at 100 °C separated by purge steps of argon with a flow rate of 100 sccm. The process consisted of 0.4 s pulse DEZ, 30 s exposure, and 40 s purge with dry argon and a 2 s pulse (H_2_O), 30 s exposure and 40 s purge. One thousand ALD cycles were carried out in order to achieve the deposition of ZnO of ≈200 nm with a growth per cycle of 0.2 nm [39]. 

Next, the sample has been characterized using scanning electron microscope (SEM, FEI S50, Hillsboro, OR, USA) to determine both geometrical properties and surface topography of the deposited ZnO layer on the microsphere. Raman spectroscopy was performed to analyze the structure of the layer on the surface of the sphere. The Raman spectra were measured by N-TEGRA Spectra (NT-MDT Company, Moscow, Russia) setup. During the experiment, the ZnO film was illuminated by a light beam generated by a laser with central wavelength λ_c_ = 532 nm. The analysis of literature shows the ZnO in wurtzite structure (bulk form) belongs to the space group C^4^_6v_ (Schoenflies notation) with two formula units per primitive cell, for which atoms occupy C_3v_ sites [40,41]. The group theory describes the Raman active zone-center optical phonon as follows: Γ_opt_ = A_1_ + 2B_1_ + 2E_2_ + E_1_ [40,41,42,43,44,45]. The literature also presents the phonons A_1_ as well as E_1_ as polar and presenting different frequencies for longitudinal-optical (LO) and transverse-optical (TO) phonons, the B_1_ mode is non-active (silent mode) [40,41,42,43,44,45].

**Figure 2:**
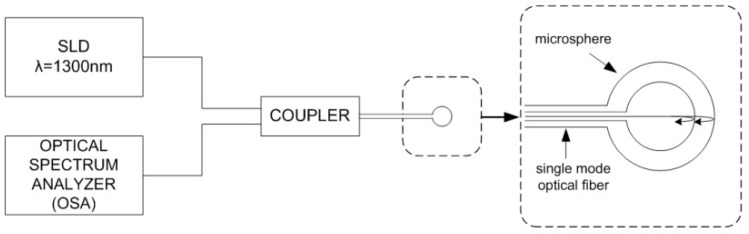
Operating principle of Fabry–Pérot interferometer used for measurements.

The microsphere was also examined using a measurement setup similar to the Fabry–Pérot interferometer operating in a reflective mode, in order to obtain information about the sensing abilities of the developed device. Operating principle of the utilized setup is shown in Figure 2 below.

The signal from a broadband superluminescent diode (SLD) light source with the central wavelength of 1290 nm and the full width at half maximum of 50 nm (S1300-G-I-20, SUPERLUM, Ireland) was applied directly to the input of a fiber-optic coupler. After reflecting on the core–cladding interface of the microsphere and the ZnO layer, the signal was detected by the Optical Spectrum Analyzer (OSA, Ando AQ6319, Kanagawa, Japan). During the experiment the sensor head was immersed in Cargille Refractive Index Liquids (Series A, AA, AAA, Cargille Laboratories, Cedar Grove, NJ, USA), each characterized by a different refractive index as follows: 1.4, 1.5, 1.6, to assess the sensing performance. Refractive indices of those liquids are provided for a wavelength of 589.3 nm. However, because of dispersion, refractive indices are accordingly lower for a longer wavelength, therefore the actual values of those liquids for a wavelength of 1300 nm are following: 1.390, 1.487, 1.576.

## 3. Theory and Calculation

Firstly, the theoretical modeling of the spectral response was performed to consider the rationale of the device. In formation of the microsphere by use of optical fiber fusion splicer, the localized melting of the fiber by arc discharge results in the creation of a spherical tip due to surface tension. The structure achieved in this process is not uniform, but exhibits the boundary of the core and cladding materials with different refractive indices. When the reflectivity of the mirrors is low, as in the investigated case, the influence of the higher order reflections in a Fabry–Pérot interferometer becomes insignificant. In addition, due to the very low thickness of the ALD film in comparison to the device structure and operating wavelength, the investigated structure can be simplified from a dual cavity interferometer (cladding layer and ZnO film) to a single cladding-material cavity interferometer with a reflection coefficient of the outermost mirror surface modeled by ZnO spectral reflection response. Therefore, the output signal of the sensor can be approximated by two-beam interferometer response—one reflected from the core/cladding interface and the second from the external surface of the microsphere and coupled back to the fiber core (Figure 1). The method for calculation of the ZnO thin film spectral reflection function was described in detail by Majchrowicz et al. [35]. The thickness of the film 200 nm was chosen for the application, as it provides highest reflection value for wavelengths in the 1300–1500 nm range [35].

The field distribution of the light propagated in the fiber core, and thus the first reflected beam, can be approximated by Gaussian distribution [46]. The transformation of the second beam, propagated in the cladding sphere, can be modeled by ray-matrix approach for the Gaussian beam. The ABCD matrix for the microsphere can be written as:

(1) $$M=\left[\begin{array}{cc} 1 & 0\\ \frac{n_{2}-n_{1}}{n_{2}R_{in}} & \frac{n_{1}}{n_{2}} \end{array}\right]\left[\begin{array}{cc} 1 & L\\ 0 & 1 \end{array}\right]\left[\begin{array}{cc} 1 & 0\\ \frac{-2}{R_{ex}} & 1 \end{array}\right]\left[\begin{array}{cc} 1 & L\\ 0 & 1 \end{array}\right]\left[\begin{array}{cc} 1 & 0\\ \frac{n_{1}-n_{2}}{n_{1}R_{in}} & \frac{n_{2}}{n_{1}} \end{array}\right]$$

where *n*_1,2_ are the refractive indices of the core and cladding, *L* is the length of the cavity, *R_in,ex_* is the radius of the core and cladded spheres.

In the case of the second interfering beam, it is also important to take into account the coupling coefficient of the reflected wave propagated in the cavity back to the optical fiber core. It can be estimated using the complex beam parameters calculated using M matrix elements and initial beam properties [47]. A detailed description of the theoretical background used for the simulation has been described [48].

## 4. Results

### 4.1. Characterization of the ZnO ALD Coating

**Figure 3:**
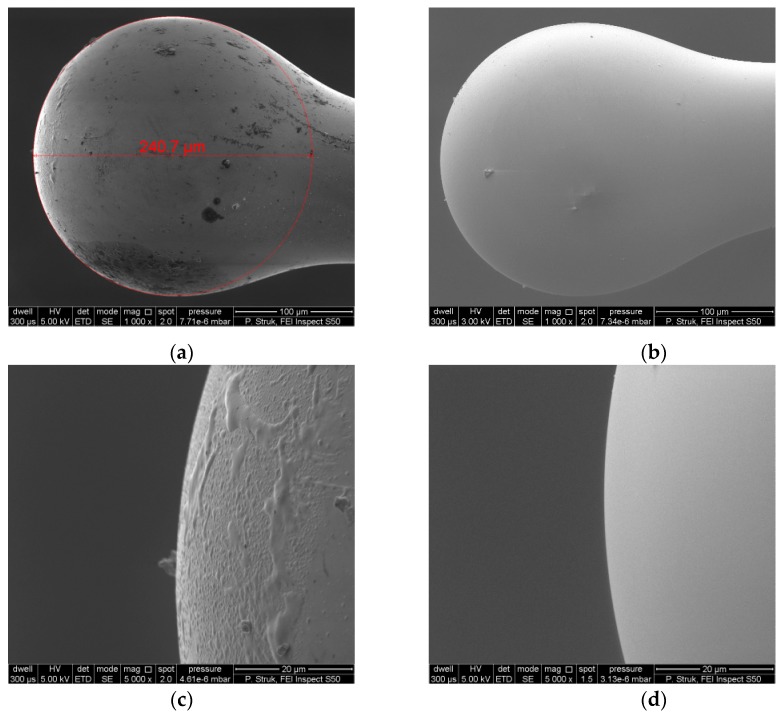
SEM images of optical fiber microsphere: (**a**) the microsphere with 200 nm ZnO coating, (**b**) the microsphere without ZnO coating, (**c**) surface topography on the front side of the microsphere with 200 nm ZnO coating, (**d**) surface topography on the front side of the microsphere without ZnO coating.

First, the morphology of the device has been examined by scanning electron microscopy (SEM). The presented microsphere shows a regular spherical shape and the diameter of the microsphere head was calculated by circle fitting and it is equal to Φ_rZnO_ = 240.7 µm for the microsphere with the ZnO layer. The SEM micrograph of the fiber-optic microsphere with deposited ZnO layer measured with 1000x magnification is presented in Figure 3a. Figure 3c shows the same surface topography on the front side of the microsphere head, measured with 5000x magnification. The ZnO layer is clearly visible. In addition, the diameter of optical fiber which was used for fabricating the microsphere was also measured and it is equal to Φ_r_ = 127.8 µm (core with cladding). In the case of the microsphere without ZnO coating, the surface topography is presented in Figure 3b,d. 

**Figure 4:**
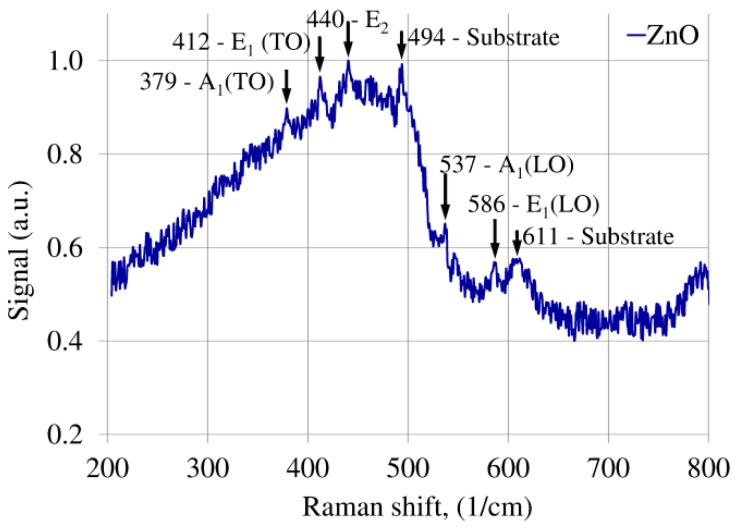
Raman spectra of the ZnO layer deposited on microsphere.

The investigation of the ZnO thin film deposited on the microsphere was carried out by Raman spectroscopy method. The Raman spectra of the ZnO film deposited on the microsphere is presented in Figure 4. The analysis of Raman peaks is: 379 cm^−1^ corresponds to acoustic mode A1 transverse-optical (TO) phonon, 412 cm^−1^ corresponds to E1 transverse-optical (TO) phonon, 440 cm^−1^ corresponds to E2, 537 cm^−1^ corresponds to A1 longitudinal-optical (LO) phonon and 586 cm^−1^ corresponds to E1 longitudinal-optical (LO) phonon. The Raman peaks for 494 cm^−1^ and 611 cm^−1^ correspond to substrate (fiber cladding–fused silica). It should be mentioned that the presented Raman peaks of ZnO are hardly visible, which can be caused by surface curvature (Φ_rZnO_ = 240.7 µm) of the microsphere. In this case, the generated Raman signal is highly diffused sideways (in comparison to a flat surface), which means that only a small part of the Raman signal can reach the detector. The presented Raman spectra confirm the ZnO layer has been deposited properly and exists on the microsphere.

### 4.2. Modeling

**Figure 5:**
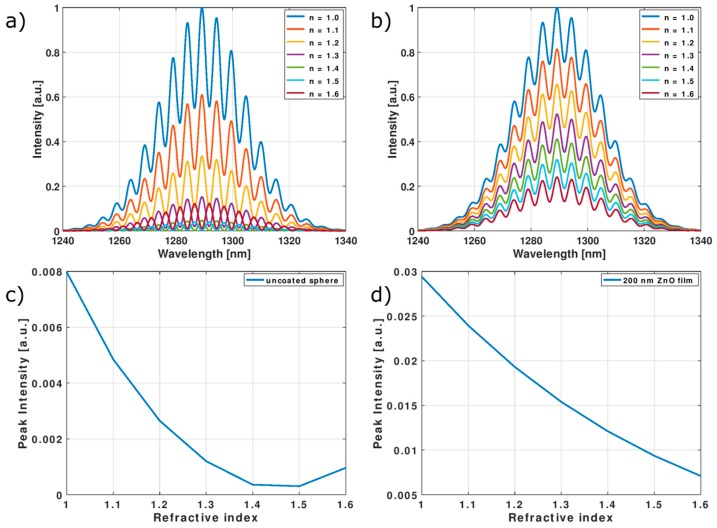
Simulation results upon illumination of superluminescent diode (SLD)-like light source: Normalized reflected spectra (**a**) for uncoated microsphere and (**b**) with 200 nm ZnO coating and signal peak intensity in function of external refractive index (**c**) for uncoated sphere and (**d**) with ZnO film.

In Figure 5a,b normalized reflected spectra of a sphere with and without ZnO film for different values of surrounding refractive index are presented. Sensor response was simulated assuming illumination with a broadband source of Gaussian characteristic; source parameters were set to λ = 1290 nm and a full width at half maximum (FWHM) ≈ 40 nm, which gives good approximation of a superluminescent diode (SLD) spectra, peak intensity was assumed as 1. The sphere geometry was as follows: *R_ex_* = 120 µm, *L* = 110 µm, *R_in_* = 10 µm. The refractive index of the fiber core was assumed as 1.46 and for the cladding 1.454 in the investigated wavelength range. Figure 5c,d show dependence of peak intensity of the output signal in function of external refractive index. As can be observed, modulation of source spectra by interference fringes is present, however the visibility of the interference signal is not high. The expected intensity of the signal obtained with the ZnO coated sphere is four times that for the clean sphere. As the external refractive index increases, the peak intensity of the reflected signal diminishes, reaching the minimum in the range between 1.4 and 1.5 for the uncoated sphere. Application of a ZnO thin film allows this extremum to shift to higher refractive index values, thus extending available sensing range to over 1.6.

### 4.3. Measurement Results

In order to determine the spectral response of the device and to evaluate the potential performance in refractive index sensing applications, a series of measurements were performed. During the experiment, the measurement head was immersed in media characterized by different refractive indices (1, 1.390, 1.487, and 1.576). 

**Figure 6:**
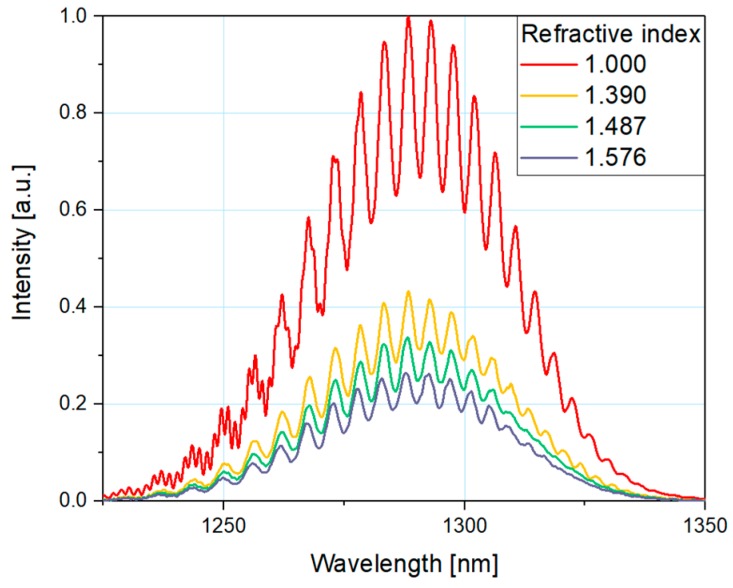
Measured response of the fiber-optic interferometer for a microsphere with ZnO layer.

**Figure 7:**
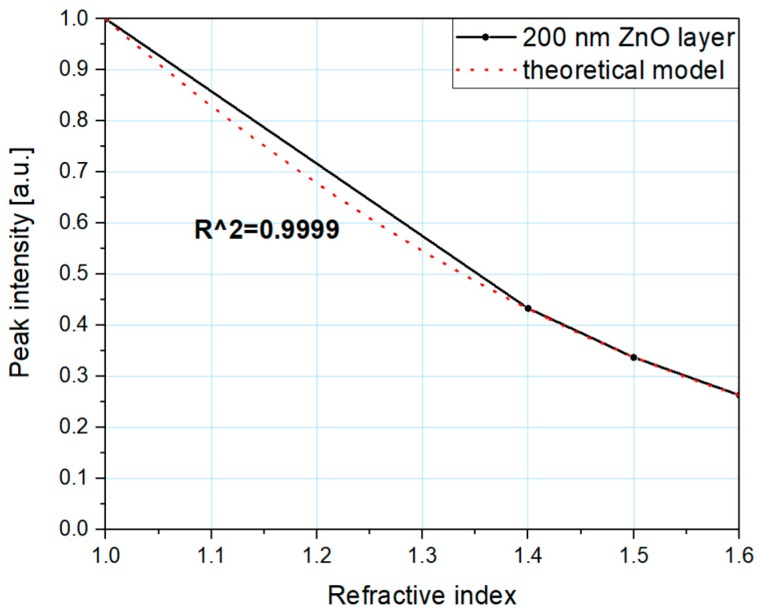
Measured response of the fiber-optic interferometer for a microsphere with ZnO layer.

In Figure 6, the measured response of the fiber-optic sensor with microsphere coated with ZnO thin film can be seen. The dependence of the signal intensity on the refractive index of the surrounding medium is shown in Figure 7. For each refractive index, the values of peak intensity were normalized to the reference value (highest value for n = 1). Theoretical modeling was included to facilitate comparison of the simulation and experimental results. Coefficient of determination, R-squared, indicates the accuracy of the measurements is close to 1 (0.9999).

It can be observed that this plot similarly traces the modeled characteristics presented in Figure 5b. The intensity of a measured signal from the sensor with ZnO coated microsphere decreases with the increase of the refractive index. The results of the experiments are in close agreement with the theoretical modeling. The signal is easily detectable in the full investigated range (up to 1.6), showing that the sensing range of a fiber-optic refractive index sensor can be successfully extended by application of a ZnO thin film coating. The modulation of reflected signal due to the interference in the microsphere structure is also clearly visible in the whole range. The density of interference fringes is not dependent on the external medium, therefore it can be used for remote checking of the integrity of sensor structure.

## 5. Conclusions

In this paper, the interferometric fiber-optic sensor with the incorporation of a microsphere coated with a thin film of ZnO by ALD at the tip of the optical fiber is reported for the first time. The sensor was first characterized using scanning electron microscope and Raman spectroscopy, confirming the presence of a conformal 200 nm ALD ZnO film around the microsphere. Theoretical simulations as well as experimental measurements have been performed to assess the performance of the device as a refractive index sensor. The measurements carried out confirmed its successful use as an optical sensor, and the enhanced sensing abilities when the microsphere was coated with the ZnO layer. Compared to other refractive index sensors reported in literature, this design allowed the measurement range to be extended. The application of a ZnO layer at the surface of a microsphere allowed us to perform measurements for refractive indices close to those of an optical fiber (1.4). Furthermore, the application of the sensor with the ALD modified microsphere allowed us to observe the interference fringes coming from the microsphere, which worked as a two-beam interferometer operating in a reflective mode. The innovative results presented in this paper open new perspectives for the sensing community and will promote the use of fiber-optic sensing devices.

## Abbreviations

ZnO	zinc oxide
ALD	atomic layer deposition
SEM	scanning electron microscopy
